# Pathogenome comparison and global phylogeny of *Escherichia coli* ST1485 strains

**DOI:** 10.1038/s41598-022-20342-0

**Published:** 2022-11-02

**Authors:** Ahmed M. Hammad, Narjol Gonzalez-Escalona, Amira El Tahan, Nasser H. Abbas, Sara S.K. Koenig, Anna Allué-Guardia, Mark Eppinger, Maria Hoffmann

**Affiliations:** 1 Department of Food Hygiene and Control, Faculty of Veterinary Medicine, University of Sadat City, Sadat City, Egypt; 2 Center for Food Safety and Applied Nutrition, U.S. Food and Drug Administration, College Park, MD USA; 3 Department of Environmental Biotechnology, Genetic Engineering and Biotechnology Research Institute, University of Sadat City, Sadat City, Egypt; 4 Department of Molecular Microbiology and Immunology, South Texas Center for Emerging Infectious Diseases (STCEID), San Antonio, TX USA

**Keywords:** Computational biology and bioinformatics, Microbiology, Molecular biology, Diseases

## Abstract

*Escherichia coli* ST1485 strains belong to the clinically important phylogroup F and have disseminated worldwide in humans, animals, and the environment. Here, we elucidated the pathogenome of a global collection of *E. coli* ST1485 isolates from diverse sources retrieved from public databases and a high-quality sequenced complete genome of colistin-resistant *E. coli* strain CFSAN061771 isolated from raw milk cheese which designated as a reference strain. CFSAN061771 belongs to O83:H42-ST1485 pathotype and carries a conjugative ColV plasmid, pCFSAN061771_01, combining extraintestinal virulence genes (*ompt*, *sitA*, *iroN*, *etsC*, *traT*, *cvaC*, *hylF*, *iss*, *tsh*, *mchf*, *iucC*, *iutA*) with a multidrug resistance island (*bla*_TEM-1_, *aph*(*6*)*-Id*, *aph*(*3″*)*-Ib*, *sul2*, *dfrA14*). Comparative genomic analysis revealed a high frequency of pCFSAN061771_01-like plasmids in *E. coli* ST1485. A notable evolutionary genetic event in *E. coli* ST1485 strains is the acquisition of a pCFSAN061771_02-like plasmid, which confers resistance to several antimicrobials, tellurium, and quaternary ammonium compounds. The identical virulence and antibiotic resistance profiles identified in some human and animal strains are worrisome. This is the first study to emphasize the significance of *E. coli* ST1485 as a global high-risk virulent and multidrug-resistant clone with zoonotic potential.

## Introduction

*Escherichia coli* is a commensal bacterium inhabiting the gastrointestinal tract of healthy humans and animals. Nevertheless, various strains have developed pathogenetic pathways capable of producing a variety of diseases. The pathogenic *E. coli* that cause diarrhea are referred to as diarrheagenic *E. coli* (DEC). They are classified into pathotypes based on their virulence characteristics and the symptoms they produce^1,2^. *Escherichia coli* strains termed extraintestinal pathogenic *E. coli* (ExPEC) exhibit a broad range of genes that encode virulence factors involved in colonization, adherence, invasion, persistence, and/or toxin production in the host, causing illnesses such as urinary tract infections, neonatal meningitis, pneumonia, and septicemia^3,4^. It is still unclear what differentiates ExPEC from commensal *E. coli*^1,2,5^. ExPEC strains mainly include uropathogenic *E. col* (UPEC), neonatal meningitis-causing *E. coli* (NMEC), avian pathogenic *E. coli* (APEC), sepsis-associated *E. coli* (SEPEC) and mammary pathogenic *E. coli* (MPEC)^4,6,7^.

Many ExPEC-associated virulence factors have been identified in the literature, either on conserved pathogenicity-associated islands (PAIs) or virulence plasmids^6^. Even though the plasmid composition of ExPEC is quite varied, virulence factors tend to be restricted to a narrow fraction of plasmid types, like ColV plasmids, which supply both virulence and fitness-associated features^8^. Such virulence ColV plasmids harbor a PAI that encodes several virulence components, including multiple iron acquisition and utilization systems, serum survival proteins, hemolysins, adhesins, outer membrane proteins, and autotransporters^9^. These virulence factors promote host colonization and invasion and dodge or impair host defensive systems causing life-threatening illnesses such as hemolytic uremic syndrome and newborn meningitis^10,11^. They are also quite prevalent among avian pathogenic *E. coli* (APEC) that cause severe respiratory and systemic disease in poultry^12^. Remarkably, ColV plasmids often have been discovered in specific ExPEC clonal groups from humans and food-producing animals, including ST95, ST131, ST117, and ST58^13–16^.

Since 2005, pan-genome analysis has been used as an efficient tool to explain the variance in genetic content among bacterial species^17^. The term “pan-genome” represents the total number of non-redundant genes in a particular dataset^18^.On the other hand, multisequence typing (MLST) has gained widespread popularity as a routine typing tool for epidemiological investigations at local and global scales^19^. It is noteworthy that a large proportion of antibiotic-resistant infections are caused by a small set of sequence types^1,20^. Indeed, the mechanism behind the remarkable worldwide spread of particular *E. coli* lineages is not fully understood, a situation that warrants regular screening of the genomic plasticity of *E. coli* isolates from different sources, particularly those emerging in animal and food of animal origin and have zoonotic importance.

*Escherichia coli* ST1485 strains belong to the clinically important phylogroup F and have disseminated worldwide in humans, animals, and the environment^21–24^. Yet to date, no genomic comparisons of *E. coli* ST1485 strains nor pan-genomic epidemiological analysis have been published. In this study, we elucidate a high-quality sequenced complete genome of *E. coli* ST1485 strain CFSAN061771, isolated from raw milk cheese in Egypt. Further, we performed comparative genomic analyses using CFSAN061771 as a reference with a global collection of *E. coli* ST1485 strains retrieved from public databases^25,26^ and clustered them based on their virulence and antibiotic resistance gene content.

## Results

### Basic genomic features

In silico genotyping revealed that *E. coli* strain CFSAN061771 belongs to ST1485, O83:H42 serogroup, and phylogroup F. The complete genome of *E. coli* strain CFSAN061771 comprises a chromosome of 4,908,204 bp with a G + C content of 50.6%. Further, two plasmids named pCFSAN061771_01 and pCFSAN061771_02 of 167,754 bp and 215,531 bp were detected, respectively. Plasmid replicons IncFIA, IncFIB (AP001918), and IncFIC (FII) were detected in pCFSAN061771_01, whereas IncHI2 and IncHI2A were detected in pCFSAN061771_02.

### Closest relative genomes to *E. coli* CFSAN061771

**Figure 1:**
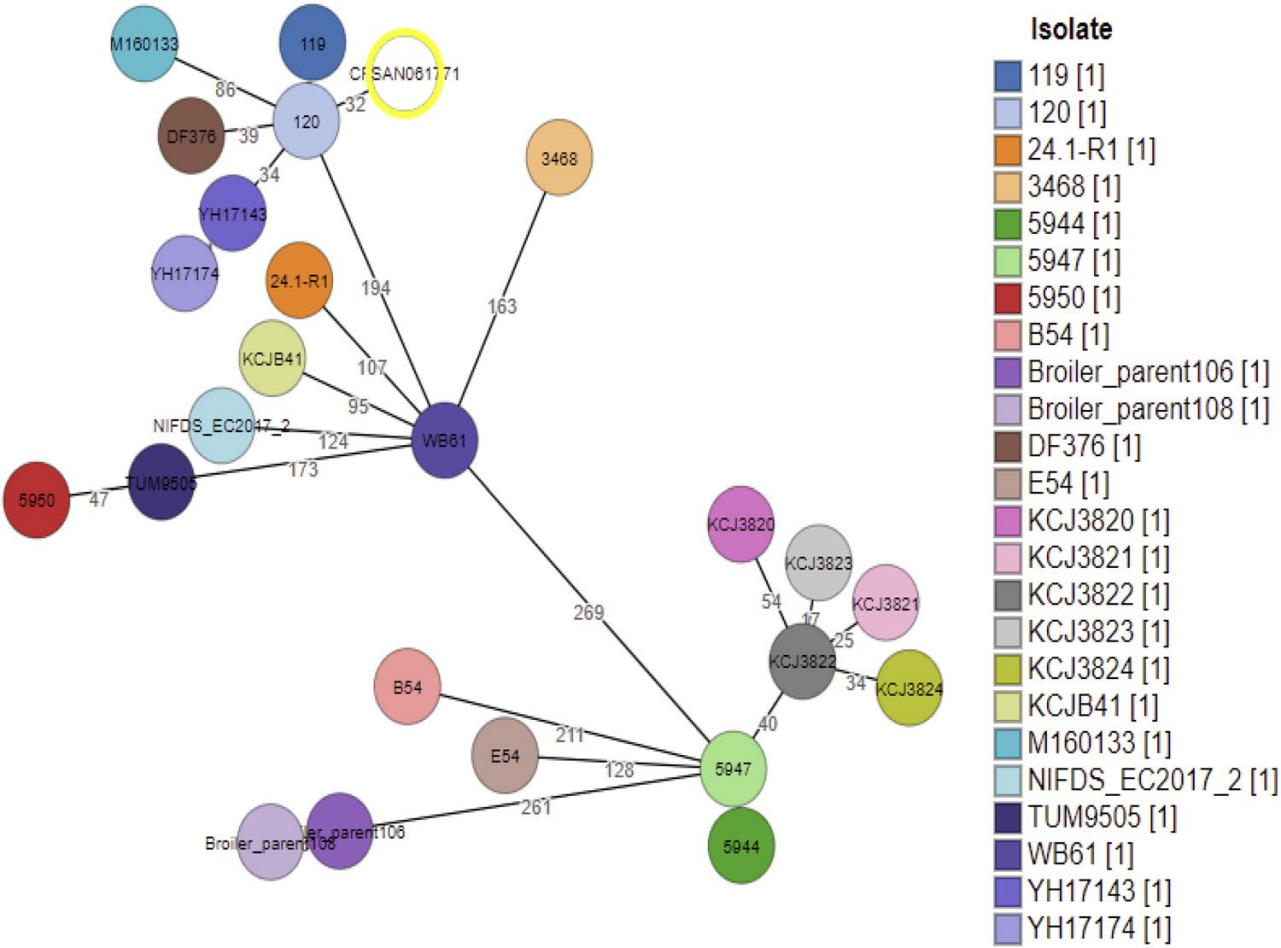
Phylogeny of closest relatives of CFSAN061771 based on cgMLST. The closest relative strains were identified by a core genome MLST (cgMLST) allele threshold of 500 alleles. CFSAN061771 is the white circle highlighted in yellow and the individual isolates are marked with different colors. The numbers on the branches represent the allelic differences.

As illustrated in Fig. 1, grapeTree was used to produce and visualize a minimum spanning tree (MST) based on comparing cgMLST allelic profiles. *Escherichia coli* CFSAN061771 was clustered with two unpublished strains, *E. coli* 119 (accession number: JABADS01) and *E. coli* 120 (accession numbers: JABADT01), isolated from ditch water in the Netherland, with only 32 allelic differences. Noteworthy, CFSAN061771 was also similar to two Chinese strains isolated from chicken, YH17143 (accession number: PTNO01) and YH17174 (accession number: PTMO01), with 39 and 43 allelic differences, respectively. The remaining two strains that were a part of the same grape are DF376 and M160133, that showed 44 and 90 allelic differences, respectively.

On the other hand, the closest reference and representative genomes to our strain identified by Mash/MinHash^27^ were YH17143, YH17174, and M160133 (accession number: CP022164) with distances of 0.00105797, 0.00115999, and 0.00216198, respectively.

### Pan-genome analysis

**Figure 2:**
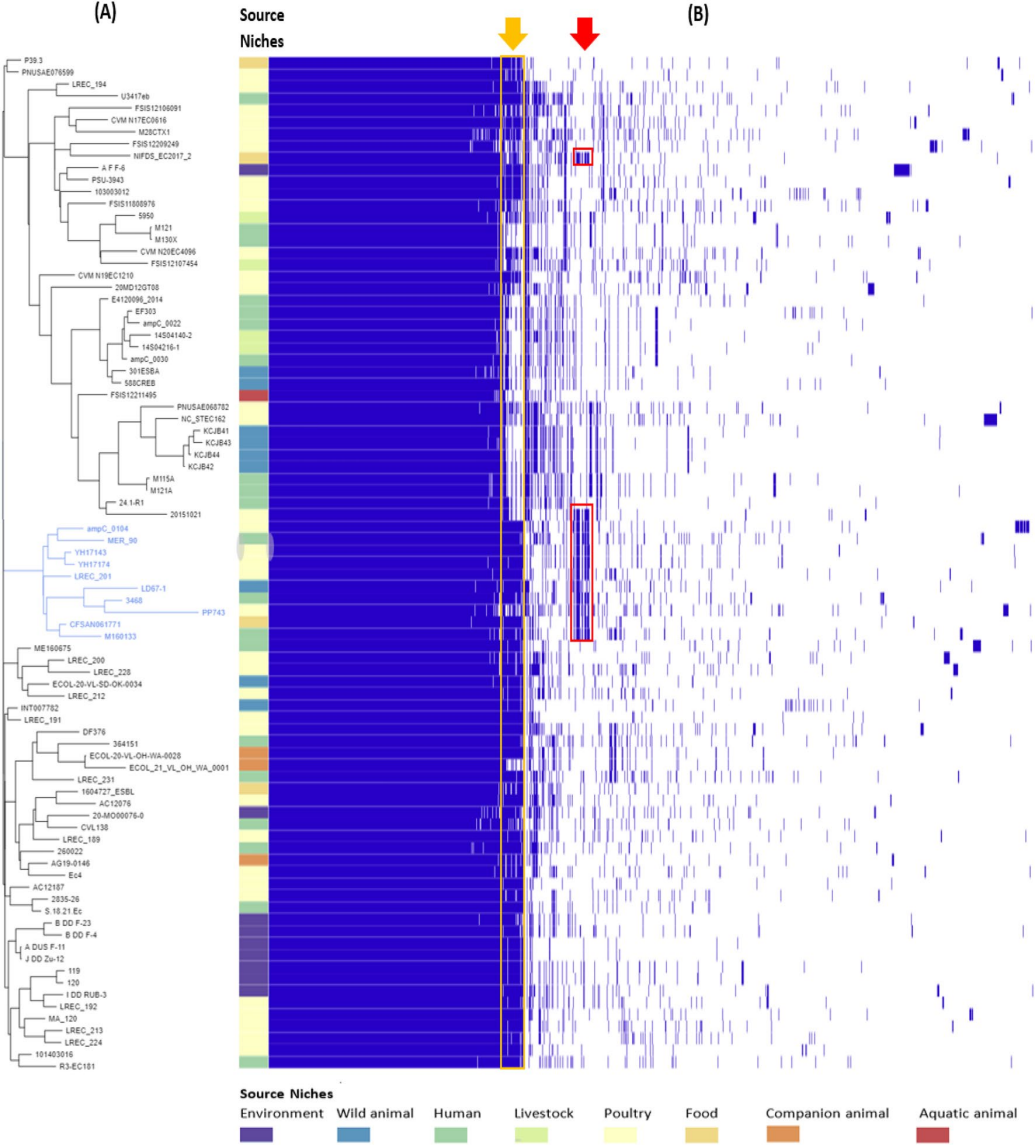
Pan-genome comparison of 85 *E. coli* ST1485 strains. (**A**) Phylogenetic tree constructed by FastTree 2 version 2.1.9^53^ based on accessory genes. (**B**) Matrix plot, which shows the presence and absence of each gene across all strains by blue and white, respectively. A complete list of annotated genes is provided in the Supplementary Table 1. Red arrow refers to the pCFSAN061771_02 like plasmid genes that are outlined by red squares, while the orange arrow refers to pCFSAN061771_01 like plasmid genes that are outlined by orange square. Source niches are denoted with different colors. All strains in the clade containing the sequenced reference strain CFSAN061771 are given in blue.

We conducted the pan-genome analysis to investigate possible differences in gene repertoires among the 85 *E. coli* ST1485 used in this study. The overall pangenome consisted of 13,376 genes represented by 3998 core genes (3527 hard + 471 soft core genes) and 9378 accessory genes (1324 shell + 8054 cloud genes). Strikingly, only 12 strains carried pCFSAN061771_02 like plasmid genes, as illustrated in Fig. 2 (outlined in red). The closest match to CFSAN061771 is M160133, a strain isolated from a human patient in the United States^23^. Pan-genome analysis revealed that they share a core genome of 4751 genes accounting for 91.1% (4751/5215) of their pan-genome. A more extensive comparison of CFSAN061771with the M160133 strain indicated that they shared 94.03% (4367/4644) of their chromosomal genes. In terms of plasmids, pCFSAN061771_01 and pM160133 p2 shared 166 core plasmid genes, accounting for 86.91% (166/191) of their pan plasmid genes, whereas pCFSAN061771_02 and pM160133_p1 share 221 core plasmid genes accounting for 78.36% (221/282) of their pan plasmid genes. Notably, the M160133 strain has an additional plasmid, pM160133_p3, consisting of 113,428 bp, not present in CFSAN061771.

### Virulome analysis

#### Chromosomal virulence genes

The chromosome of CFSAN061771 carries different virulence genes that are commonly involved in urinary tract infections, including *yfcV* (encodes the major subunit of a putative chaperone-usher fimbria), *chuA* (encodes a heme binding protein)^28^, heat-resistant agglutinin (*hra*) gene^29^, outer membrane protein (*ompT*) gene^30^, and capsular genes *(kpsE, kpsMII)*^31^*.* Interestingly, it also carries enteroaggregative *E. coli* (EAEC) virulence gene regulator (*eilA*), *air* gene that encodes enteroaggregative immunoglobulin repeat protein^32^, and long polar fimbriae (*lpfA*) gene that was detected in EAEC and other pathogenic *E. coli* strains^33^. Furthermore, it carries *ompA, ibeB, ibeC*, and *aslA* genes that encode structures critical to neonatal meningitis *E. coli* (NMEC) for crossing of the blood–brain barrier and subsequently invasion of brain endothelial cells^34,35^. It also harbors two bacteriocins, *mchF* and *mcmA*, which encode for the ABC transporter MchF and the microcin M, respectively, and are associated with antibacterial activity against closely related species^36^.

#### Plasmid virulence genes

**Figure 3:**
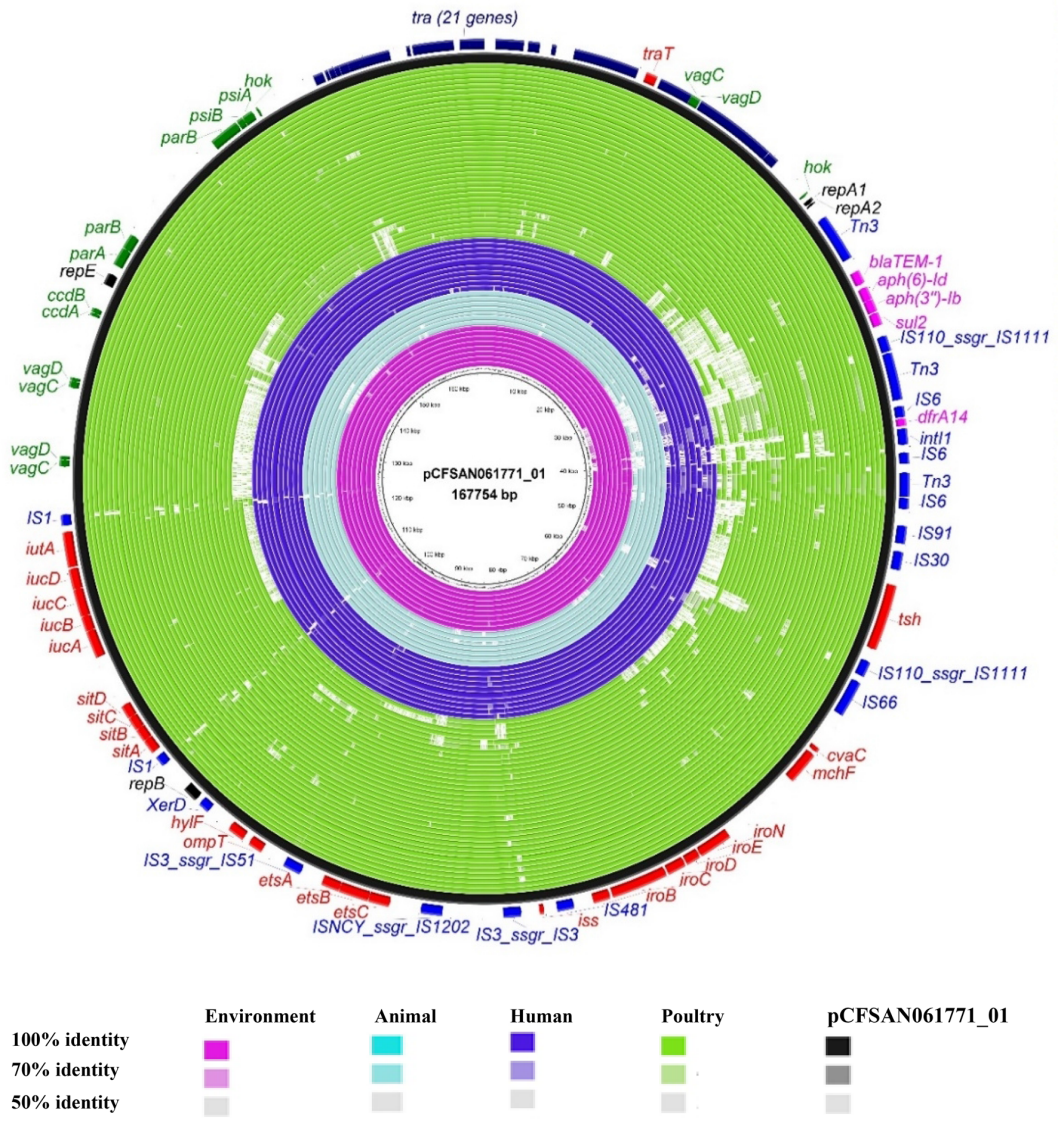
BRIG comparison of pCFSAN061771_01–like plasmids in *Escherichia coli* ST1485 strains. The pCFSAN061771_01 plasmid from the CFSAN061771 strain was used as reference for alignment and gene annotation and is shown in the outermost black circle. Query genomes are color-coded according to source and the order plotted in the circle reflects their similarities to pCFSAN061771_01. Gene inventories are colored as follows: red, virulence genes; fuchsia, antibiotic resistance genes; blue, insertion sequences; green (maintenance genes); black replication genes; navy, *tra* locus. Strains are arranged from inside as follow: environmental strains (water) A F F-6, 119, A DUS F-11, J DD Zu-12, I DD RUB-3, 120, 20-MO00076-0, B DD F-4; animal strains (livestock, wild, and companion animals), FSIS12107454, ECOL-20-VL-SD-OK-003, 45,950, ECOL-20-VL-OH-WA-0028, AG19-0146, LD67-1, INT007782; human strains, ME160675, MER-90, S.18.21.Ec, 34 68, M160133, LREC_23, 1U3417eb, 364,151, 260,022, R3-EC181; poultry strains, 20MD12GT08, NC_STEC162, PNUSAE068782, CVM N20EC4096, CVM N19EC1210, FSIS11808976, PNUSAE076599, FSIS12106091, PSU-3943, DF376, M28CTX1, YH17174, YH17143, ampC_0104, 2835-26, MA_120, 103,003,012, CVM N17EC0616, 101,403,016, Ec4, PP743, FSIS12209249, LREC_201, AC12187, LREC_200, AC12076, LREC_213, LREC_192, LREC_224, LREC_212, LREC_194, LREC_191, LREC_228, LREC_189.

The genetic organization of the virulence genes in pCFSAN061771_01 is presented in Fig. 3. The plasmid carries the genes encoding: colicin V (ColV operon) (*cvaABC*, *cvi*); a core region of ColV plasmids which includes three different iron uptake and utilization systems (ferric aerobactin system (*iutA/iucABCD*), iron and manganese ABC transport system (*sitABCD*), and salmochelin siderophore system (*iroBCDEN*)); an outer membrane protein T-encoding gene *ompT*; ABC transport system *etsABC*; the increased serum survival gene involved in complement resistance *iss*; and a hemolysin-encoding gene *hlyF*. The putative virulence region of pCFSAN061771_01 also harbors *tsh* gene, which encodes temperature-sensitive hemagglutinin that was confirmed to be associated with the virulence of APEC^37^. Additionally, different maintenance systems associated with virulence plasmids were identified, including plasmid partitioning system (*parABS*) and toxin-antitoxin-based addiction systems. The toxin-antitoxin-based addiction systems comprised: postsegregational killing (PSK) system, *ccdA/ccdB*; virulence-associated genes, *vagC* and *vagD*; and host killing gene, *hok*^38^. Further, the whole transfer (*tra)* region (*traN*, *traF*, *traQ*, *traH*, *traG*, *traT*, *traD*, *traI*, *traX*, *traJ*, *traY*, *traA*, *traL*, *traE*, *traK*, *traB*, *traP*, *traR*, *traC*, *traW*, and *traU*) that encodes for the transfer components of plasmids were detected. On the other hand, pCFSAN061771_02 did not carry any virulence genes.

#### The global spread of pCFSAN061771_01 and pCFSAN061771_02 like plasmids in *E. coli* ST1485

**Figure 4:**
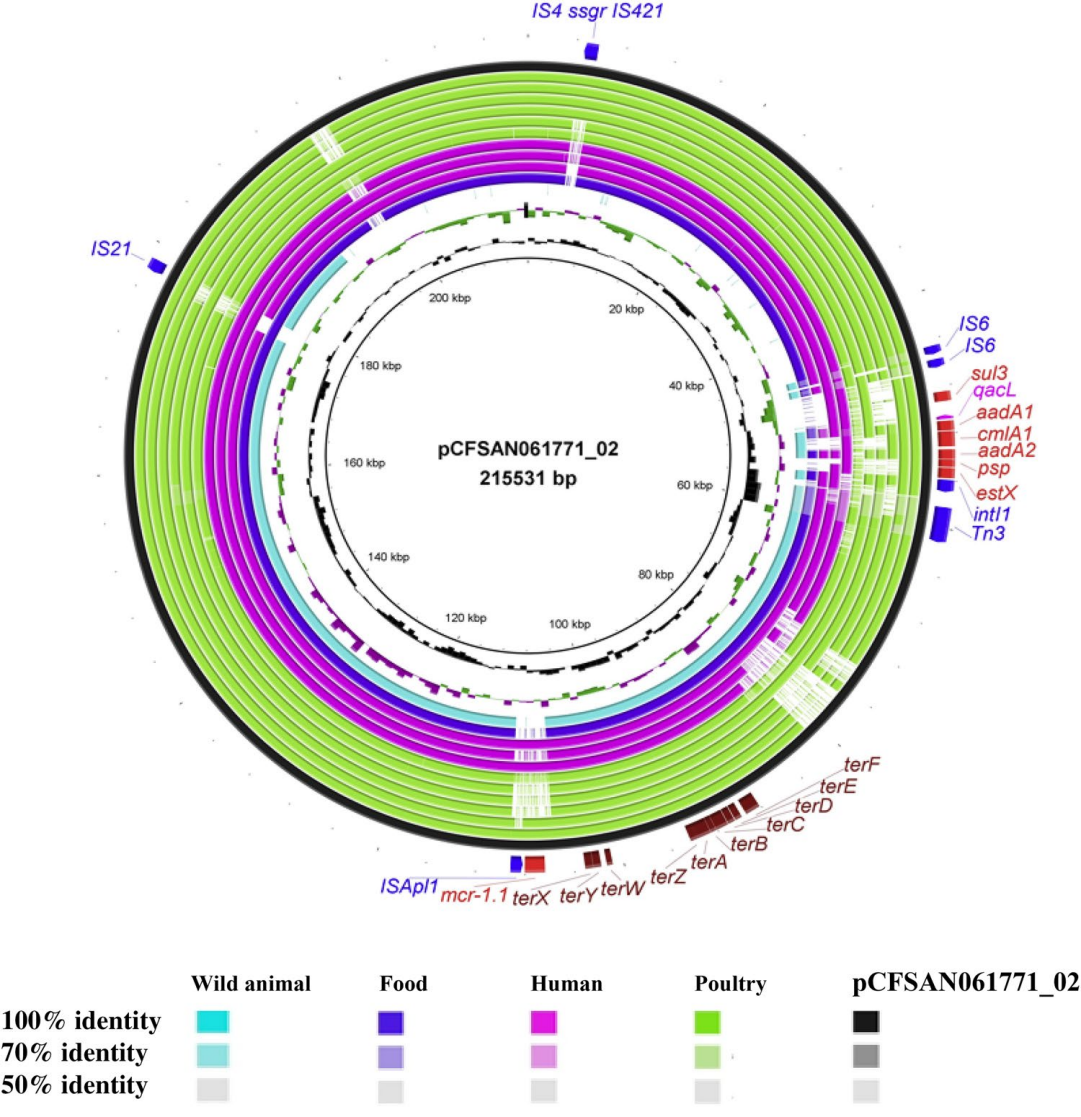
BRIG comparison of pCFSAN061771_02–like plasmids in *Escherichia coli* ST1485 strains. The pCFSAN061771_02 plasmid from the CFSAN061771 strain was used as reference for alignment and gene annotation as is shown in the outermost black circle. Query genomes are color-coded according to source and the order plotted in the circle reflects their similarities to pCFSAN061771_02. Gene inventories are colored as follow: red (antibiotic resistance genes; blue (mobile elements); fuchsia (quaternary ammonium compound); and maroon (*tar* locus). Strains are arranged from inside as follow; animal strain LD67-1 (pLD67-1-157 kb); food strain (NIFDS_EC2017_2); human strains (M160133 (pM160133_p1), MER_90, 3468); poultry strains (PP743, LREC_201, ampC_0104, 20,151,021, YH17174, YH17143).

The alignment of contigs of 84 genomes retrieved from public databases on the pCFSAN061771_01 sequence further confirmed the presence of pCFSAN061771_01–like plasmids in most *E. coli* ST1485 strains, regardless of country and source of isolation (Fig. 3). On the other hand, when we used pCFSAN061771_02 as a BLAST query sequence against 84 *E. coli* ST1485 genome sequences, only 11 genomes were returned. These genomes were identified from: animal, LD67-1 (pLD67-1-157 kb); food (NIFDS_EC2017_2); human (M160133 (pM160133_p1), MER_90, 3468); and poultry (PP743, LREC_201, ampC_0104, 20,151,021, YH17174, YH17143) (Fig. 4).

#### Clustering of globally disseminated *E. coli* ST1485 strains based on their virulence profiles

**Figure 5:**
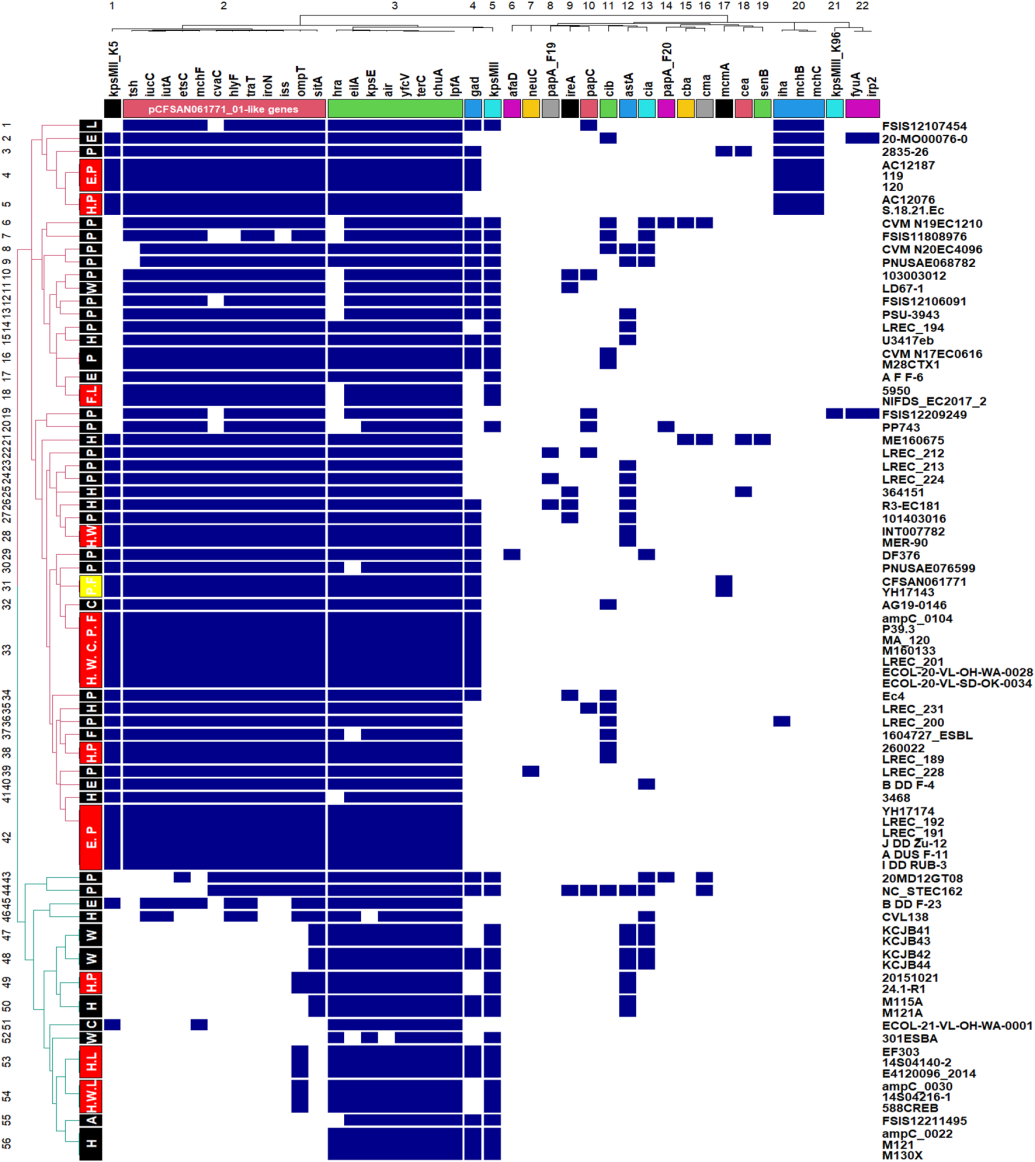
Heat map demonstrating the distribution of virulence genes in *E. coli* ST1485 strains. Blue represents the presence and white represents the absence of a virulence gene. Strains from various origins with identical virulence profiles are denoted as red boxes on the left, whereas strains from the same source were denoted as black boxes. The yellow box denotes the sequenced strain CFSAN061771 (cluster 31). *H* human, *L* livestock, *P* poultry, *C* companion animals, *W* wild animals, *F* food, *A* aquatic animal.

Figure 5 shows that 85 *E. coli* ST1485 strains, including CFSAN061771, harbored seven or more virulence genes from a panel of 43 genes and displayed 56 virulence patterns. It is worth noting that the majority of strains from diverse sources had eight or more genes of a panel consisting of 12 virulence genes (*ompt*, *sitA*, *iroN*, *etsC*, *traT*, *cvaC*, *hylF*, *iss*, *tsh*, *mchf*, *iucC*, *iutA*) grouped in cluster 2, shown in red, which is likewise present on pCFSAN061771_01. All strains harbored at least six genes from another panel comprised of eight genes grouped in cluster 3, which was shown in green (*hra*, *eilA*, *kpsE*, *air*, *yfcv*, *terC*, *chuA*, *IpfA*). Our food strain (marked as a yellow box on the left of Fig. 5) clustered with a poultry strain (YH17143) with the same virulence pattern.

### Resistome analysis

#### Chromosomal antibiotic resistance genes

The chromosome of CFSAN061771 possesses mutations in the DNA gyrase (*gyrA*, S83L) and *parC* (S80I), which are associated with fluoroquinolone resistance.

#### Plasmids' antibiotic resistance genes

**Figure 6:**
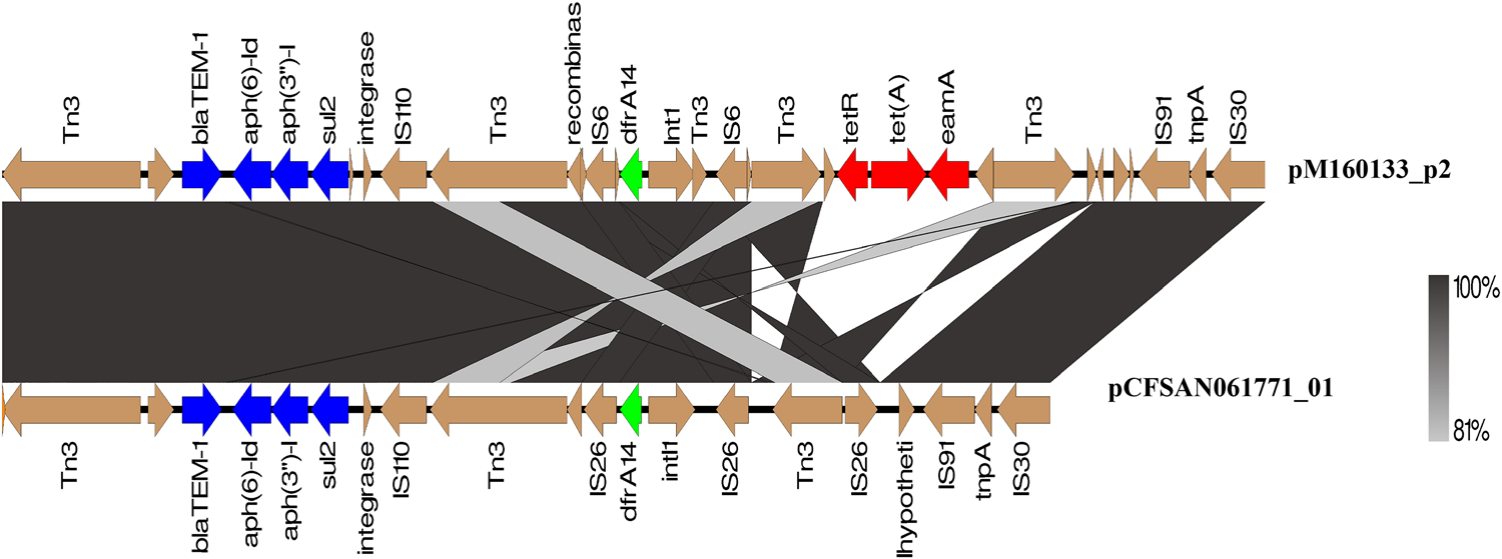
Linear maps of the multidrug resistance regions of pCFSAN061771_01 and pM160133_p2. Antimicrobial resistance genes are shown in red, green and blue. Mobile elements are shown in brown. Homologous segments with ≥ 99% sequence identity are indicated by black shading, while gray shading shows inverted homologous segments.

pCFSAN061771_01 harbors an MDR-encoding region integrated with several mobile elements (Fig. 3). It comprises a cluster of genes encoding resistance to sulfonamide (*sul2*), β-lactam (*bla*_TEM-IB_), kanamycin (*aph*(*3″*)*-Ib*), and streptomycin (*aph*(*6*)*-Id*) flanked by *tnpR* and IS*110* as well as a class 1 integron (Int191) harboring a sole gene cassette, *dfrA14*, encoding resistance to trimethoprim. The genetic organization of this region in our strain and pM160133_p2, a plasmid of closely related strain M160133, were compared in Fig. 6. Notably, CFSAN061771 lacks the tetracycline *tet*A. On the other hand, as shown in Fig. 4, pCFSAN061771_02 harbored *mcr-1* gene, encoding colistin resistance with an upstream copy of IS*Apl1* as well as class 1 integron, In641, with gene cassettes encoding resistance to aminoglycosides (*estX*, *aadA2, aadA2)*, chloramphenicol (*cmlA1*) and quaternary ammonium compounds (*qacL*). The sulfonamide resistance *gene, sul3,* which was found to be associated with In64^39^, was also detected. The genetic organization of antibiotic resistance genes and alignment of genome sequences on the pCFSAN061771_02 sequence is illustrated in Fig. 4.

#### Clustering of globally disseminated *E. coli* ST1485 strains based on their antibiotic resistance profiles

**Figure 7:**
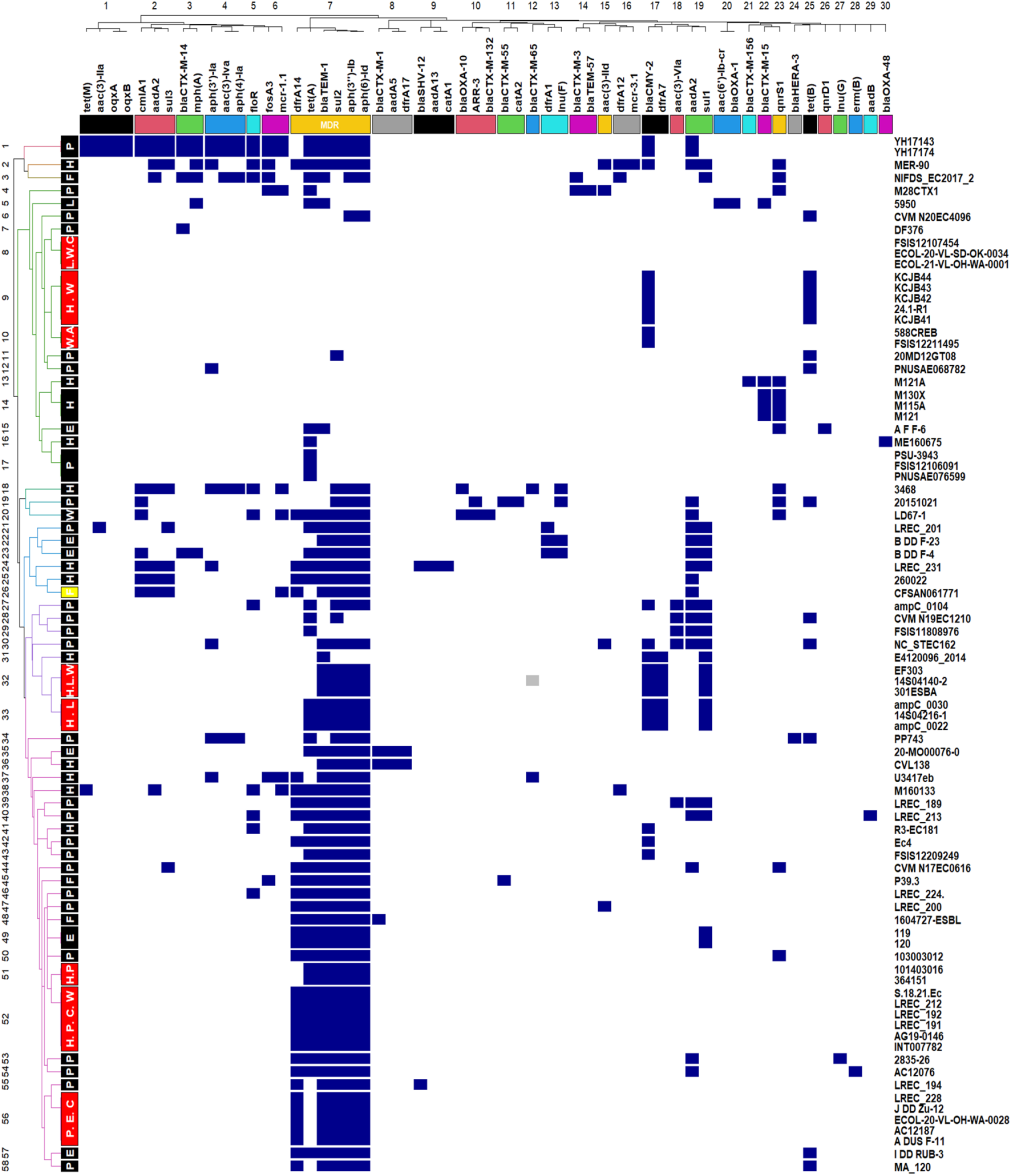
Heat map demonstrating the distribution of antibiotic resistance genes in *E. coli* ST1485 strains. Blue represents the presence and white represents the absence of a virulence gene. Strains from various origins with identical virulence profiles were denoted as red squares on the left, whereas strains from the same source were denoted as black squares. The yellow square denotes the sequenced reference strain CFSAN061771. *H* human, *L* livestock, *P* poultry, *C* companion animals, *W* wild animals, *F* food, *A* aquatic animal.

Figure 7 shows that 85 *E. coli* ST1485 strains harbored one or more antibiotic resistance genes from a panel of 57 genes and displayed 58 resistance patterns. Antibiotic resistance genes were clustered into 30 clusters, and those in cluster 7 (*bla*_TEM-1_, *aph*(*6*)*-Id*, *aph*(*3″*)*-Ib*, *sul2*, *dfrA14*) were found to compromise a multidrug resistance region (MDR) in pCFSAN061771_01 (Fig. 6). Our strain formed a separate cluster (cluster 26) and was shown as a yellow box on the left of Fig. 7.

## Discussion

The availability of high-quality annotated complete genome sequences of potentially pathogenic *E. coli* from non-clinical sources provides a crucial resource for understanding the pathogenome evolution among different *E. coli* pathotypes. In this study, we elucidated the pathogenome of a global collection of *E. coli* ST1485 isolates from diverse sources retrieved from public databases and a high-quality sequenced complete genome of colistin-resistant *E. coli* strain CFSAN061771 isolated from raw milk cheese in Egypt which designated as a reference strain. Notably, few dominant STs are thought to make up the population of phylogroup F isolates which are characterized by a high prevalence of virulence and antibiotic resistance genes (such as ST59, ST354, ST405, and ST648)^40^. Here, the importance of evolving *E. coli* ST1485 belonging to phylogroup F as a worldwide high-risk virulent and multidrug-resistant clone is highlighted.

Pan-genome analysis with virulence and antibiotic resistance profiling revealed that CFSAN061771 was related to the urinary *E. coli* strain isolated from a New York patient, suggesting its zoonotic potential^23^. It is essential to point out that the cgMLST demonstrated a lesser resolving ability than the pan-genome analysis did when it came to grouping closely related strains that had comparable or identical virulence and antibiotic resistance characteristics. This is because the cgMLST tree was constructed using predefined core genes of the individual strains without their accessory genes^41^. It is worth noting that epidemiological studies that rely on publicly available genetic data are sometimes uncertain as to whether the whole dataset accurately represents the genomic and source variation of the entire population. Even though poultry made up most of our source population, we think we have observed most of the genomic diversity in ST1485 because of its large pangenome, and wide range of antibiotic resistance and virulence genes.

The identification of ColV plasmids in literature is based on Liu’s criteria which consider a strain to be ColV-positive if it possesses at least one or more genes from four or more of the six gene sets (i) *cvaABC* and *cvi*, (ii) *iroBCDEN*, (iii) *iucABCD* and *iutA*, (iv) *etsABC*, (v) *ompT* and *hlyF*, and (vi) sitABCD^13^. But these criteria are limited because they only take into consideration specific genes and don't consider the architecture of the plasmid backbone. Therefore, in order to test the theory that ColV plasmids were responsible for the evolution of ST1485 as a pathogen, we analyzed a global collection of 84 draft genomes for the presence of closed ColV plasmid sequence pCFSAN061771_01. ST1485 strains have acquired a diversity of ColV plasmids, and most of the ColV plasmids studied contain the complete repertoire of archetypal ColV genetic backbone. Figure 3 displays the evolutionary changes in ColV-like plasmid sequences in *E. coli* ST1485 strains recovered from diverse sources. Collectively, poultry strains show the highest striking differences in gene repertoire of ColV pCFSAN061771_01 like plasmids. The extraordinary consistency of ColV plasmid carriage across most of the *E. coli* ST1485 strains from diverse origins and geographical regions may be attributable to the presence of multiple plasmid maintenance systems that allow sustained plasmid inheritance, even in the absence of selection pressure^38^. The evidence points to the possibility that *E. coli* ST1485 might serve as a potential reservoir for disseminating this virulence and multidrug resistance plasmid.

A notable evolutionary genetic event in *E. coli* ST1485 is the acquisition of pCFSAN061771_02-like plasmid, which confers selective advantages for several antibiotics (aminoglycosides, chloramphenicol, sulfonamides, colistin), tellurium, and quaternary ammonium compounds. Colistin has become the last-resort antibiotic for treating lethal infections caused by multidrug-resistant *Enterobacteriaceae* in human medicine. The World Organisation also classified it as a Veterinary Highly Important Antimicrobial Agent (VHIA) for Animal Health^42^. Considering the relevance of human movement and migration in the rapid transcontinental transfer of antibiotic-resistant bacteria, the continuous availability of colistin over-the-counter in certain nations remains a public health concern.

Interestingly, the constructed phylogenetic tree based on pan-genome analysis demonstrated that 10 out of 12 strains carried a pCFSAN061771_02-like plasmid clustered in one clade, including a food strain (CFSAN061771), an animal strain (LD67-1), human strains (M160133, MER_90, 3468) and poultry strains (LREC_201, ampC_0104, PP743, YH17174, YH17143) (Fig. 2). This finding reflects a significant genetic overlap among human, animal, and poultry *E. coli* ST1485 strains from many regions throughout the globe, raising concerns about the zoonotic potential of *E. coli* ST1485. Furthermore, using pCFSAN061771_02 as a reference, a comparison of draft genomes harboring the pCFSAN061771_02-like plasmid as well as two fully sequenced plasmids (pLD67-1-157 kb from LD67-1 and pM160133_p1 from M160133)^23,24^, indicated backbone conservation (Fig. 5).

It is surprising how identical the virulence and antibiotic resistance profiles are in some humans (H), livestock (L), food (F), poultry (P), companions (C), and wild animals (W) (denoted as red boxes on the left annotation of Figs. 5 and 7). The insertion of MDR-encoding island in pCFSAN061771_01-like plasmids carrying extraintestinal virulence genes is especially concerning. This MDR island is rich in mobile elements and operates as a hotspot for sequential gene uptake through multiple mechanisms (Fig. 6). It seems to be an essential feature of *E. coli* ST1485's adaptive machinery for colonizing various niches. Our findings point toward that *E. coli* ST1485 can adapt to several hosts armed with either pCFSAN061771_01 or pCFSAN061771_02- like plasmids or both, which confers selective advantages for the host strain, contributing to the recent emergence of *E. coli* ST1485. We also detected *E. coli* ST1485 strains devoid of pCFSAN061771_01 and pCFSAN061771_02's virulence and antibiotic resistance genes, which might be the ancestor of *E. coli* ST1485 from which these hybrid genotype strains were formed.

UPEC has a variety of structural and secreted virulence elements that contribute to their ability to cause illness, including flagella, fimbriae, pili, toxins, and iron-acquisition systems. They were defined as strains positive to two or more *chuA* loci (heme-binding proteins), *fyuA* (encodes the yersiniabactin receptor), *vat* (encodes an autotransporter serine protease toxin), and *yfcV* (encodes the major subunit of a putative chaperone-usher fimbria)^28^. According to these criteria, all the *E. coli* ST1485 strains studied, including CFSAN061771, have likely the potential to be uropathogens as they carried both *chuA* and *yfcV* (Fig. 5). The emergence of the virulence gene ferric yersiniabactin uptake receptor (*fyuA*) in an environmental strain (20-MO00076-0) and a poultry strain (FSIS12209249) implies the propensity of *E. coli* ST1485 strains to acquire more virulence genes. On the other hand, the carriage of iron acquisition systems on the ColV plasmids identified in this study are crucial for UPEC survival in an iron-limited environment such as the urinary tract, providing additional virulence potential for *E. coli* ST1485 strains to cause urinary tract infection. Worryingly, *traT* gene, which was reported to be harbored exclusively on IncF plasmids and involved in increasing resistance to serum killing, was found in *tra* operon of pCFSAN061771_01 and pCFSAN061771_01 like plasmids. This gene was frequently detected in UPEC^43^ and NMEC strain from France^44^. Interestingly, a recent study revealed that it was also a prevalent gene in *E. coli* strains isolated from dairy cows with clinical mastitis^45^, raising the concern about the possible dissemination of this gene through consumption of contaminated dairy products. This work demonstrates that the foodborne *E. coli* ST1485 strain has the potential to function as a uropathogen, stressing the urgent need for more research to identify the percentage of extraintestinal illness in humans caused by *E. coli* ST1485 acquired through food.

In conclusion, the deciphering of the pathogenome of the emerging hybrid *E. coli* ST1485 strain, CFSAN061771, and screening of the virulence profiles of globally disseminated *E. coli* ST1485 strains from various sources, reveals the outstanding ability of this sequence type belonging to the phylogroup F to acquire virulence genes implicated in both intestinal and extraintestinal pathogenicity. *Escherichia coli* ST1485 is a potential reservoir of ColV plasmids, and the carriage of ColV plasmids is not generally present nor exclusive to APEC strains; rather, they have emerged in strains from various sources, including environment, animal, and human isolates. Some strains of this sequence type, including CFSAN061771, have exceptional versatility in integrating newly discovered genes such as *mcr-1* and genes conferring broad antibiotic resistance. The notion that the *mcr-1* gene has made its way into *E. coli* ST1485- a sequence type capable of colonizing different hosts- could make it hard to prevent further transmission of this element to humans. The identical virulence and antibiotic resistance profiles identified in some human and animal *E. coli* ST1485 strains are worrisome. They suggest that this sequence type may have zoonotic potential, posing a serious threat to public health, and should be closely monitored across the world. Finally, this study provides new insights into the extent and possibly human health implications of consuming raw milk cheese which may be a vehicle for disseminating cattle-associated hypervirulent and multidrug-resistant *E. coli* ST1485 to humans, causing multiple diseases, particularly urinary tract infections.

## Methods

### Bacterial strain

In a study conducted from December 2016 to February 2017 aimed to determine the incidence and molecular characteristics of colistin-resistant *E. coli* in karish cheese, a famous Egyptian raw milk cheese, we cultivated colistin-resistant *E. coli* strains from two hundred samples (100 g each of karish cheese). At the Department of Food Hygiene and Control, Faculty of Veterinary Medicine, University of Sadat City, Egypt, 225 ml of buffered peptone water (Oxoid, Basingstoke, England) were added to cheese samples (25 g) in a sterile plastic package. The samples were subsequently homogenized in a stomacher, and spread onto plates of eosin methylene blue agar (Oxoid, Basingstoke, England) containing 2 mg/l colistin. Then the plates were incubated for 24 h at 37 °C. Colonies grown on eosin methylene were tested by traditional biochemical tests. Strains showed typical *E. coli* phenotypes by the API 20E system (bioMe'rieux, Marcy l'Etoile, France) and were shipped to the Center for Food Safety and Applied Nutrition, U.S. Food and Drug Administration, College Park, Maryland, USA. Upon arriving at the FDA laboratory, the CFSAN061771 *E. coli* strain was chosen for sequencing.

### DNA extraction and genome sequencing of CFSAN061771

The genomic DNA of strain CFSAN061771 was isolated from overnight culture using the DNeasy Blood and Tissue kit (Qiagen Inc., Valencia, CA) and sequenced on the Pacific Biosciences (PacBio) RS II sequencing platform as previously described^46^. The sequences were annotated using the NCBI Prokaryotic Genomes Annotation Pipeline (PGAP)^47^.

### Publicly available sequences

Enterobase (http://enterobase.warwick.ac.uk/species/index/ecoli)^25^ and PATRIC (https://www.patricbrc.org/)^26^databases were queried for released ST1485 whole-genome sequences with available metadata for source, collection year, continent, and country. Associated metadata for these sequences is available in Supplementary Table 1.

### Genomic analyses

CFSAN061771’s genome was in silico typed with regard to O:H serotypes and sequence type by the database of the Center for Genomic Epidemiology (https://cge.cbs.dtu.dk/services/) using SerotypeFinder^48^, and MLSTtyper^49^. BacWGSTdb (http://bacdb.cn/BacWGSTdb/^50^was used to determine the closest relative strains with the function “Single genome analysis” and a core genome MLST (cgMLST) allele threshold based on 500 alleles. The “Similar genome finder” service in PATRIC (https://www.patricbrc.org/)^26^ was used to find the closest reference and representative genomes to our strain based on Mash distance^27^**.**

### Pan-genome analysis

The genome of CFSAN061771 and a global collection of 84 genomes were annotated using Prokka^51^, and the generated gff3 files were used as input for pangenome identification with Roary^52^, which groups full-length genes into core (hard and soft core) and accessory (shell and cloud) genomes. Hard-core genes are present in > 99 percent of genomes, whereas soft-core genes are found in 95–99 percent of genomes. Shell and cloud genes exit in 15–95% and less than 15% of genomes, respectively. This analysis yielded a matrix of core and accessory gene presence/absence. The phylogenetic tree was generated using FastTree 2^53^ based on the presence or absence of accessory genes. The matrix of presence/absence genes was then compared to the phylogenetic tree and visualized using Phandango^54^.

### Screening for the presence of similar sequences to CFSAN061771’s plasmids

*E. coli* ST1485 genomes retrieved from public databases^25,26^ were investigated for the presence of similar sequences to pCFSAN061771_01 and pCFSAN061771_02 using nucleotide-nucleotide BLAST^55^. Similar sequences were visualized with Blast Ring Image Generator (BRIG)^56^.

### Analysis of plasmid replicons, virulome, resistome, and mobilome

The presence of antibiotic resistance and virulence determinants and plasmid replicons were screened using ABRicate v0.8.10^57^ (https://github.com/tseemann/ABRicate) with the ResFinder database^58^, Virulence Factor Database (VFDB)^59^, and PlasmidFinder database^60^ (cutoffs, identity, and coverage of > 90%). Chromosomal mutations associated with antimicrobial resistance genes were studied using PointFinder available at https://cge.cbs.dtu.dk/services/ResFinder/^58^. ISfinder^61^ was used to identify insertion sequences and transposons bracketing antibiotic resistance and virulence genes. Additionally, we screened 84 strains of a global collection of *E. coli* ST1485 for their virulence and antibiotic resistance gene content by using BLASTN against previously described reference genes in VirulenceFinder and ResFinder databases^6,58^. ComplexHeatmap (v2.6.2) R package^62^ was used to plot a summary heatmap for the presence or absence of virulence and antibiotic resistance genes.

## Supplementary Information

Supplementary Information.

## Data Availability

All data generated or analyzed during this study are included in this published article and in the Supplementary Table 1. The complete nucleotide sequences of *E. coli* strain CFSAN061771 chromosome pCFSAN061771_01 and pCFSAN061771_02 have been deposited into GenBank under the accession numbers of NZ_CP042896, NZ_CP042897, and NZ_CP042898, respectively.
